# MicroRNA and Cardiovascular Disease 2016

**DOI:** 10.1155/2017/3780513

**Published:** 2017-03-27

**Authors:** Ling-Qing Yuan, Vinicio de Jesus Perez, Xiao-Bo Liao, Magdalena Król, Chi-Hsiao Yeh

**Affiliations:** ^1^Department of Metabolism and Endocrinology, The Second Xiangya Hospital, Central South University, Changsha 410011, China; ^2^Division of Pulmonary and Critical Care Medicine, Stanford University Medical Center, Stanford, CA 94305, USA; ^3^Departments of Cardiothoracic Surgery, The Second Xiangya Hospital, Central South University, Changsha 410011, China; ^4^Warsaw University of Life Sciences, 02-787 Warsaw, Poland; ^5^Chang Gung Memorial Hospital, Keelung 20445, Taiwan

A decade of research and development has shed light on the multifunctional characteristics and clinical importance of microRNA in cardiovascular diseases (CVDs). More and more evidence supports important roles of microRNAs in the pathogenesis, diagnosis, and potential treatment for various CVDs. This special issue includes several interesting studies and reviews on microRNA and CVD that aim at broadening the translational and clinical research frontiers in this exciting field.

Translational medicine aims at accelerating the implementation of basic science discoveries into clinical practice. The review by E. Cavarretta and G. Frati entitled “MicroRNAs in Coronary Heart Disease: Ready to Enter the Clinical Arena?” provides a systematic review on how miRNAs could serve as biomarkers on common CADs such as acute coronary syndromes and myocardial infarction. Their discussion includes consideration of the current technical challenges preventing their implementation in clinical practice, such as how to normalize miRNA values, how drugs could affect clinical measurements, and the range of values to be used in practice. While it is clear that miRNA-based therapy has great potential for CADs, there is still a long way to go before this becomes a reality.

Also, in this issue, X. Lin et al. have provided a review entitled “Function, Role, and Clinical Application of MicroRNAs in Vascular Aging,” in which the role of microRNAs in the regulation of vascular aging is summarized, with a focus on how they impact the function of endothelial and vascular smooth muscle cells. Indeed, microRNAs are involved in modulating cell differentiation, proliferation, migration, senescence, apoptosis, and angiogenesis, all of which play critical roles in the pathogenesis of vascular aging. Furthermore, the potential application of microRNAs to clinical practice for the diagnosis and treatment of cardiovascular diseases is also discussed.

Epicardial adipose tissue, the metabolically active visceral fat around the heart, was proven to be associated with CADs and metabolic disorders. Y. Liu et al.'s study entitled “Role of miRNAs in Epicardial Adipose Tissue in CAD Patients with T2DM” used a microarray-based approach to identify microRNAs differentially expressed in epicardial adipose tissue in CAD patient with T2DM. Eleven microRNAs were selected for validation and their target genes were predicted using computational methods, which suggested that the insulin signaling pathway is potentially involved in the pathogenesis of CAD and metabolic disorders. Their study proposes that dysregulation of microRNAs in EAT might be associated with the pathogenesis of CAD and T2DM.

The study by A. Krajewska et al. entitled “Paroxysmal Atrial Fibrillation in the Course of Acute Pulmonary Embolism: Clinical Significance and Impact on Prognosis” analyzes the relationship and clinical implications between atrial fibrillation (AF) and acute pulmonary embolism (PE). They found that paroxysmal AF may be a sign of PE severity and may affect long-term prognosis. Their explanation is that sudden increase in right ventricular pressure results in a concomitant increase in right atrial pressure, leading to atrial tachyarrhythmia. Moreover, the author also found that PE patients with AF have lower risk of deep vein thrombosis (DVT) compared with patients in normal sinus rhythm.

Y. Ding et al.'s review entitled “MicroRNAs and Cardiovascular Disease in Diabetes Mellitus” addresses the state of basic research and clinical studies on the contribution of microRNAs to CAD associated with diabetes mellitus. Abnormal expression of microRNAs induced by hyperglycemia is involved in endothelial cell injury, proliferation of VSMCs, platelet adhesion, and macrophage and lipid accumulation. The latter could trigger atherosclerosis and increase the morbidity and mortality of CAD in diabetes mellitus. Thus, microRNAs and their related gene targets could be potential biomarkers and therapeutic targets of CVD in patients with diabetes.

We hope these researches and reviews will bring new insights and spark ideas for future research and inspire readers who wish to pursue studies in the growing field of microRNA and CVD.

*Ling-Qing Yuan* *Vinicio de Jesus Perez* *Xiao-Bo Liao* *Magdalena Król* *Chi-Hsiao Yeh*

